# Reasons to care: Personal motivation as a key factor in the practice of the professional foster carer in Romania

**DOI:** 10.1371/journal.pone.0222716

**Published:** 2019-09-18

**Authors:** Alexandru Neagoe, Doina Larisa Maria Neag, Daniel Lucheș

**Affiliations:** 1 Department of Social Work, The West University of Timișoara, Timișoara, Romania; 2 Department of Sociology, The West University of Timișoara, Timișoara, Romania; Middlesex University, UNITED KINGDOM

## Abstract

Personal motivation is a key factor in the service of foster care, impending both on the welfare of the child and on the satisfaction of the carer. This paper explores the benefits, challenges and dilemmas involved in the job of professional (i.e. state-supported) foster carer in Romania–a country where the issue of child protection has drawn a great deal of international attention over the last thirty years. The principal hypothesis concerns whether the benefits, challenges and dilemmas identified by foster carers are influenced by the factors that led to their taking up this kind of work. Quantitative research was conducted using a questionnaire as the main tool. The paper takes a descriptive, cross-sectional and multifactorial approach. Sampling was carried out by self-selecting method, and the study involved 51 participants. The research project identified a statistically significant variation in the challenges and dilemmas reported by foster carers. Thus, the results of the study show that the majority of the carers indicate a primarily intrinsic motivation for their work. By way of conclusion, it is argued that social workers, operating in collaboration with multidisciplinary teams, can offer carers support in managing more difficult periods in the child–carer relationship, thus enhancing the sustainability of the foster care service.

## Introduction

The issue of personal motivation in the practice of foster care has received significant scholarly attention in recent years [1–5]. As indicated by many of the existing studies, the specifics of foster care services depend heavily on the local cultural and social milieu. A particularly significant case, in this respect, is that of Romania, for several reasons. First, the collapse of the communist regime in Romania, at the end of 1989, has brought a lot of international attention to the magnitude and complexity of problems being faced by the children from the state-run orphanages of that time [6]. Second, despite a lot of important social changes in Romania over the last three decades, the number of children in need of special care continues to be very high, due to a wide range of factors, but in particular due to the work migration phenomenon associated with the income gap between Romania and Western Europe [7–8]. Third, at a cultural level, Romania stands out as one of the most religiously-oriented societies in Europe [9], with the implication that social care services (including foster care) are often intertwined with religious factors—mostly related to the Christian religion [10]. Given this overall situation, the issue of foster care in Romania (and especially the issue of personal motivation in foster care) is a significant one not only at regional or national level but also for the international academic community.

According to Romanian Ministry of Labour and Social Justice statistics, as published in their report for the last quarter of 2017, there were 55,302 children in the special protection system during that period, of whom 18,197 were in residential units and 37,105 were in family-type foster care. Of this last group, 18,421 were in the care of professional foster carers [11] (p. 2).

During recent years, Romanian legislation on professional foster care has (by Government Decision 625/2000) made compulsory an initial evaluation of potential foster carers. The questions that those wishing to become foster carers have to answer during this evaluation include ones about their motivation for deciding to pursue this kind of work. It is, thus, understood that the carers’ personal motivation can play a significant role in what may be regarded as a sustainable foster care service–one which is able to facilitate a healthy dynamic between all the other factors which impend on the child’s welfare (social, psychological, educational, etc.). Moreover, given the crucial role which foster carers play in the education and development of the children whom they care for, as well as the close connection between people’s motivation and their personal values, our discussion about foster carers’ motivation will also touch on the issue of their personal values and the values they may wish to transfer towards the children.

### Extrinsic and intrinsic motivation

Specialised literature classifies sources of motivation into two categories, extrinsic and intrinsic [12].

Someone’s motivation can be described as extrinsic when its source lies not in the person themselves but in something external [13] (p. 446). *Extrinsic motivation* is aimed at satisfying some indirect need, for example for money. In a career context, extrinsic motivation arises from a wish to meet a need such as paying for a holiday, purchasing a car, or buying a house, or for meeting other needs that cost less but still have to be paid for. Where extrinsic motivation is concerned, a person’s job is simply a means of satisfying certain needs via their salary [14] (p. 8).

*Intrinsic motivation*, on the other hand, comes from within one's self; is internalized in the individual’s value system [15]. Some would argue that it can also refer to the enjoyment which someone finds in the actual activity they engage in. People who carry out particular activities for the sake of the satisfaction they experience and not in order to gain a reward with which they can fulfil other needs can be described as being led by an “inner voice”. Where intrinsic motivation is concerned, fulfilling a task which is regarded as being enjoyable is the most important (and internally determined) reward [16] (pp.10-11).

In the realm of foster care, the motivation that has led to people deciding to become carers is extremely important, since the quality of the service they offer can depend on it. Although the majority of foster carers mention altruism and a love for children as what motivates them, there are also some who are motivated externally by the salary they receive and who become foster carers because they need a job. It is only right to record that external motivation may change to internal in the course of a carer’s work. As a result of the attachment that grows up during the period when they are looking after a child, a foster carer may reach the stage of providing services and doing things not for the sake of the salary they will receive at the end of the month but out of the attachment and affection involved in their relationship with the child [17] (p. 10).

### Money as a factor in the motivation of professional foster carers

According to the March 2018 report of the Bihor county Social Work and Child Protection Authority, a professional foster carer receives a gross monthly salary of 2,620 Romanian lei (approx. 560 €), in the context in which the average monthly salary in Romania at the time was getting close to 1,000 € (i.e. approx. 600€ net salary; [18]. Bonuses for caring for a disabled child and for a second child could increase this to a maximum gross salary of 3,153 lei (approx. 675 €), but not all foster carers qualify for that sum. When compared to remuneration in other EU countries (or even average wages in Romania), it is clear that foster carers in Romania are poorly paid for the work they do.

A recent increase in the number of foster carers who have no blood relationship with the children concerned has caused the institutions and NGOs which provide child protection services to pay close attention to the motivation of people who want to become foster carers. Those who opt to become foster parents report doing so from a range of motives, most often based on altruism and a sense of social responsibility. The majority of those who become foster carers are drawn to this work by the idea of an obligation to society and by a desire to make a difference to a child’s future. Other motives include a wish to meet a social need, religious reasons, wanting to increase one’s income, the view that fostering is a first step towards adopting, a desire to increase the number of people in one’s family, and wanting to replace a child lost through death [19] (p. 83).

Many studies have concluded that the additional income earned by carrying out the work of a foster parent is not usually the main reason why a person becomes a professional foster carer.

More specifically, a recent US study of foster families has shown that economic motives and those related to values are equally important. This would suggest that extrinsic and intrinsic types of motivation both influence foster carers to a similar extent [4] (p. 9).

### Moral values subscribed to by professional foster carers

Emil Durkheim, regarded by many as the founder of sociology, developed the theory of social solidarity to highlight the cohesion that exists between individuals. According to him, every person has a sense of social responsibility formed over the course of their life and influenced by the culture, environment and family in which they have lived. This social responsibility is formed from the sum of the beliefs and values held by the majority of the members of society. In societies in which solidarity is a very major feature, the individual no longer belongs to himself but has to do what society asks of him [20] (pp. 45–46).

Moral values, like other values, are desirable qualities but frequently it appears that they need to be even more than that, since, for example, being good rather than bad is not merely desirable but actually an obligation. Morality in itself is not an option or an individual preference but is compulsory [21] (pp. 13–15).

The assumption that there will be an acquisition of values via the upbringing provided by the foster carer is evident from the fact that the DGASPC (General Headquarters of Social Work and Child Protection) evaluation of foster carers includes a moral evaluation of them and of the other members of the family the child will be joining. A future foster carer must have positive social relationships in their local context and be able to provide evidence that they have not been found guilty of offences that would disqualify them from working as foster carers [22] (p. 30). Generally speaking, the members of carers’ families have a very significant part to play in the promotion of values and the upbringing of foster children [23] (p. 64). This moral responsibility of foster carers is also in line with the recognised need for supervision among professionals working in social services [24] and with the specific skills and qualities which are expected from those who offer education and care in a professional context [25].

## Materials and methods

### Purpose, objectives, variables and design

The overall purpose of this research project was the identification of the positive aspects, challenges and dilemmas encountered by professional foster carers in Romania as they care for and bring up their foster children.

The specific objectives are subordinate to this purpose. The first is the identification of the positive aspects, challenges and dilemmas that the foster carer encounters in their relationship with the child in their charge. The second is the identification of the value professional foster carers place on the moral and Christian formation of the child for whom they are caring.

The following hypotheses were formulated:

1. Foster carers whose motivation for taking up this kind of work is intrinsic in nature are more affected by situations in which their foster children refuse to follow their moral and Christian training than those motivated extrinsically.
2. The positive benefits, challenges and dilemmas identified by foster carers differ in accordance with the kind of motivation that led them to take up this profession.
3. The challenges encountered by foster carers in their work of caring for children are affected by the age of the child and by the carer’s age, the background from which they come, how long they have been fostering, and their religious beliefs.
4. There is an association between the positive benefits a foster carer identifies in their work and the strength of their desire that their moral and Christian values should be adopted by their foster children.

This quantitative study had the aim of analysing both similarities between the dependent variables and differences between the ways the dependent variables were influenced by the independent ones.

The *independent variables* were the environment (urban or rural) from which the foster carer came, their age, their religious beliefs, how long they had been doing this work, and their professed motivation (extrinsic or intrinsic).

The *dependent variables* were as follows:

- perceived benefits (providing a moral example, providing a spiritual example, being involved in shaping the character of a child, forming special relationships, encouraging a child to maximise their potential, other benefits)
- reported challenges (disciplining the child, coping with rejection from the child, changing negative behaviours, inadequate attachment, alcohol/drug/tobacco abuse, children who experience bullying, other challenges)
- reported dilemmas (ethical, those involving the prospect of separation from the child, religious, other dilemmas)

The research project was descriptive in character, and of a cross-sectional and multifactorial kind.

### Participants

The target group for this study was made up of professional foster carers employed by the Social Work and Child Protection Directorate. All participants live in Bihor county and were contacted with the support and encouragement of the Bihor Social Work and Child Protection Directorate. Under the aegis of the Directorate, identical training sessions are held on three consecutive days in every month, in order to give all the professional foster carers in the county the opportunity to arrange to attend on one of those three days. In May 2018 we received a positive response to our request to be allowed to carry out a study within and with the support of the Directorate. According to data available on the official Bihor DGASPC site, in May 2018 there were 376 professional foster carers on the payroll, with 654 children in their charge.

The participants in the quantitative study (N = 51) were all women, with 16% between the age of 18 and 35; 25% between 36 and 45; 57% between 46 and 55; and 2% over 56. Of the 51 participants, 42 had between 11 and 20 years’ experience as professional foster carers; 4 between 6 and 10; 3 between 1 and 5; and 2 had over 20 years of experience. 49% of participants belonged to the Orthodox religious denomination, 39.2% to the Romanian Evangelical Alliance (Pentecostals, Baptists and Brethren) and the rest to other denominations (Roman Catholic, Reformed, Seventh Day Adventist and others). In terms of the environments from which the carers came, most (70.6%) were from rural and only 29.4% from urban areas. The children in placement with the professional foster carers who participated in the study were aged from five months to 19 years.

### The research tool employed

The research method employed was of a quantitative kind. A 16-item questionnaire was administered (see Supporting information at the end of this article), with the first question asking participants to choose one from a list of 11 possible reasons why they opted to become foster carers. This question and its possible answers were formulated along the lines of research carried out by James Barber and Paul Delfabbro [17].

The following five items were questions designed to discover the positive opportunities, challenges and dilemmas encountered by foster carers looking after children. These were followed by five questions that explored the importance attached by the carer to the cultivation of Christian and moral values in their work of bringing up the children in their charge. The final items of the questionnaire were devoted to personal information. These were deliberately placed at the end in order to avoid giving the impression of an uncongenial “interrogation”. All the items in the questionnaire were designed to correspond closely with the proposed research objectives.

The *procedure* employed in the study involved the following method of selecting participants. Study participants were selected from a total population of 376 people who worked as foster carers in 2018 in Bihor County, Romania. The entire reference population was invited by DGASPC representatives to participate in a training session for one of three set days (24th, 25th or 26th May 2018). The total number of people attending these training sessions was 124, out of which 55 people showed willingness to complete the data collection tool. Following the validation of the instruments that were applied, a total of four questionnaires were removed from the analysis (due to insufficient completion). Thus, the total number of respondents whose answers were included in the study was 51. The average time taken to complete the questionnaire was eight minutes. It should be also specified that the respondents' participation in this study was voluntary, with subjects being able to quit at any time during the completion of the questionnaires. The subjects were information on the anonymity and confidentiality of the collected responses.

We appreciate that the sample of the 51 participants is representative, the percentage of respondents related to the total reference population being 13.6% (response rate). The findings of the study can be, therefore, regarded as representative for the reference population of foster carers in the county of Bihor, Romania, for the year 2018.

### Ethics statements

Before the research commenced, Alexandru Neagoe, on behalf of the authors, wrote to “The Committee for Ethics and Academic Deontology of the West University of Timisoara”, asking approval for conducting their research (Registration no. 1079 / 02 May 2018). The request letter included a general description of the research (i.e. research title, research summary, ethical implications for human subjects, study method, procedure, and expected results), as well as the fact that a written informed consent was going to be asked from the participants, before they answered the questionnaire. A sample of the written informed consent was sent to the Ethics Committee as an Annex.

The approval was granted with the registration number UVT11026 / 16 May 2018 and is available upon request.

### Data analysis

Analysis was performed using the SPSS program. Correlation of variables which showed a *symmetrical* distribution was carried out using the two-tailed Pearson correlation test, while *asymmetrically* distributed variables were analysed by means of the two-tailed Spearman rank correlation test. Difference analysis for parametrically distributed data was performed using the Independent Sample t-test while for non-parametrically distributed data the Mann-Whitney U test was applied. Results were considered to be significant only for values less than 5%.

## Results

### Results in relation to the established objectives

#### Intrinsic versus extrinsic motivation

Of all those who took part in the study, 61% had an intrinsic motivation for choosing to work as professional foster carers, while the remainder (39%) were extrinsically motivated, showing that the majority of participants chose to become professional foster carers out of an inner desire to contribute to society by providing personal support to a child who had no family or whose family could not meet their needs.

The research tool helped us to highlight the principal motivation of participants. 43.1% of participants stated that their decision to become professional foster carers was based on altruism. The second most important motivation was needing a job (17.6% of those who mentioned this were registered unemployed, 15.7% were in need of a new career and 5.9% gave various other financial reasons). Participants in this category had chosen the career of professional foster carer for the reason that at the time they needed to find work they had had no other feasible options. 16% of foster carers gave motivations based on religious convictions and said that they regarded doing this kind of work as a calling given them by God. 3.9% of foster carers had chosen this profession because they had encountered medical problems in conceiving their own child.

#### Benefits

An open-ended question gave participants the opportunity to identify other benefits that they had received from working as foster carers. 33% of the carers stated in response to this that the personal satisfaction derived from helping abandoned children was the greatest benefit they could identify. Item 3 of the questionnaire, “Please list other benefits you have experienced from working in this area”, received such responses as “giving a better life and an upbringing to someone who had limited chances in life”, “seeing him take his first steps a few months after I had taken him home with me”, “the satisfaction of having brought up and helped children who do not have the help of their biological parents” and “because I have managed to help a child and to give them a mother’s love and the affection they need at this time”.

It was interesting that 31% of respondents specified that working as foster carers had brought them such experiences as joy, love, peace, happiness, delight, surprise and other positive emotions that they would not have had otherwise. A significant figure of 22% specified that “the fact of caring for a child” is itself a major benefit conferred by professional foster care. 10% of participants stated that the profession of foster carer had brought them personal development. Preparation for adoption was given by 2% of respondents as a benefit they had experienced.

#### Challenges

Where challenges were concerned, disciplining children and changing negative behaviour patterns were seen as the most serious, each receiving a score of 39%. Foster carers found times when it became necessary to implement these two measures difficult. Their consciousness of a twofold responsibility, as employees and also as parents, probably contributed to their sense of the gravity of the challenge posed by these two problems. Challenges caused by the child experiencing bullying received a score of 12%. Other challenges faced by foster carers were rejection by the child (8%) and insecure/poor attachment on the part of the child in placement (2%).

#### Dilemmas and concerns

When we turn to dilemmas/concerns identified by foster carers, the highest percentage (33%) had to do with education and upbringing, followed by health (27%), future separation from the child (22%), ethical aspects of working in foster care (12%), and religious dilemmas related to the child (2%). 4% of respondents stated that they had not experienced any dilemmas/concerns in regard to looking after the child in placement with them.

### Hypothesis testing

This quantitative study had the aim of analysing both similarities between the dependent variables and differences between the ways the dependent variables are influenced by the independent ones.

#### Intrinsic vs. extrinsic motivation

The first hypothesis was that foster carers whose decision to undertake this kind of work sprang from an intrinsic motivation would be more affected when children in their charge rejected their moral and Christian training than carers whose motivation was extrinsic in origin. The rationale of this hypothesis was that if foster carers had a motivation that was based on altruism and civic spirit, they would naturally be more affected if a child refused to accept their training regarding values and morality. However, this hypothesis was not validated for this group of participants; the type of motivation involved had no significant measurable impact on the extent to which foster carers were affected if a child refused to accept their moral instruction.

#### Personal motivation vs. benefits, challenges, and dilemmas

The second hypothesis was expressed as follows: there would be differences between the benefits, challenges and dilemmas identified by foster carers that could be accounted for by the type of motivation that had taken them into this profession. What we found was a difference in terms of dilemmas identified by foster carers whose motivation was based on their need for a job and by those motivated by altruism, civic spirit and generosity. Both results of the Mann-Whitney U Tests were significant at the 5% level.

#### Challenges vs age, environment, experience, and religion

The third hypothesis was formulated as follows: the challenges encountered by foster carers are affected by the age of the child and by the foster carer’s age, the rural vs urban environment from which they came, how long they had been doing this kind of work, and their religious beliefs.

For this hypothesis the dependent variable was made up of the various challenges experienced by foster carers, while the independent variable was the length of their experience in this kind of work, divided into intervals representing different numbers of years’ experience. It was found that challenges identified depended on carers’ length of experience. Specifically, the study found differences between the challenges encountered by the group with between six and ten years’ experience and those reported by the group with 11–20 years’ experience. The results of the Mann-Whitney U Test were significant at the 1% level.

Thus, for this group of foster carers, the challenges encountered were not affected by the age of the child in placement or by the carer’s age, background or religious beliefs but only by the length of experience they had had of fostering.

#### Benefits vs. expectations

The fourth hypothesis was that there would be an association between the benefits foster carers identified as coming from their work and the strength of their expectation that their own values should be adopted by the children in their care. This hypothesis was confirmed by calculation of Spearman’s (rho), with a very high level of significance less than 5%. Our conclusion is that there are significant correlations between the level of benefit identified and the intensity of the participants’ expectation that their foster children should live in accordance with moral and Christian values.

## Discussion

The purpose of this study was to give a general outline impression of the complex role of the professional foster carer in Romania (a country where the issue of child protection has drawn a lot of international attention over the last thirty years). By means of a quantitative study carried out within and with the support of the Bihor county DGASPC in May 2018, valuable information was obtained regarding the benefits, challenges and dilemmas/concerns faced by foster carers, as well as about the importance attached by substitute parents to moral and Christian child-rearing.

The results of our research confirm the findings of similar studies in other parts of the world, regarding the predominantly intrinsic motivation of foster carers [1,2,4,5,16], as well as related recent research highlighting the prevalence of moral rationale, rather than external factors such as equity or efficiency, in influencing human prosociality [26–28]. Despite the significantly lower levels of income in Romania, as compared to most other EU countries, the primary declared motivation in the respondents’ decision to become foster carers is not related to extrinsic factors (although these cannot be neglected) but to their intrinsic desire to make a positive difference in a child’s life.

The study allowed us to establish that for our sample, playing a part in the forming of a character is an *opportunity* identified by the largest number of foster carers. Other significant opportunities or benefits selected included the personal satisfaction and positive emotions that flow from working in foster care.

Turning to the *challenges* involved in this profession, disciplining children and changing negative behaviour patterns were the most widely identified. *Dilemmas* connected with child-rearing and with the ethical standards that belong to the profession of substitute parent were the concerns that the highest percentages of respondents mentioned. Other significant concerns facing foster carers included handling the moment of separation from the foster child, together with the discrimination experienced by the child in society.

Although we believe the results of this study to be significant, it also has its limitations. One of these has to do with the relatively small number of participants. Another is the fact that participants were exclusively female. It is probable that this study, like many others, was affected by the “phenomenon of desirability”, i.e. respondents’ tendency to give the perceived desired answer, which would be a further limitation. The setting for the application of the questionnaire was a hallway in the DGASPC building, where the participants had to sit together at tables to complete their questionnaire forms. It is quite possible that respondents may have selected answers that “looked good” for the simple reason that there was someone near them who could read what they were writing. A further limitation affecting the study was the relatively short time period within which the 55 completed questionnaires (of which 51 were valid and usable) were obtained. Only three days were available on which data could be collected.

## Conclusions

Our research findings highlight the need to devote particular attention to foster carers and to the moment at which they become substitute parents for a new child. We have been able to see that alongside the professional benefits and satisfaction they experience, they also face challenges and dilemmas of very many kinds. It is true that (as we have shown) most say that they have become foster carers from motives of altruism, a wish to be of help to society, and out of a sense of civic spirit for their communities, but this does not fully equip them with the knowledge they will need to deal successfully with the crises ahead. The moment at which a new child comes into the foster carer’s family is a zero point from which everything begins. The child does not know the family, the family does not know the child, and both sides need to begin a process of adaptation that can often be a source of stress and emotional pressure. We would propose, therefore, the introduction of intensive sessions on “coping”, covering a number of areas such as child psychology, the upbringing of children, health, and parentcraft, to equip carers with new information that will be helpful both to them and to the foster child. At present, foster carers in Romania are legally entitled to some initial training, with the opportunity to attend professional training courses and receive counselling during their period of work. We believe every foster carer and every child should be regarded as unique cases that require a tailor-made approach, which is why there is a need to create interdisciplinary teams that will be available for foster carers to call upon. In the majority of cases, an abandoned or orphan child is not happy and carefree but, sadly, someone damaged by traumas, violence and abuse. At the point at which they are entrusted to their foster carer, their memory still bears the impress of the past and they behave in ways that match the way they have been treated. It is for this reason that the negative behaviour, rejecting behaviour and need for discipline of such a child pose such a severe challenge to their substitute parents. The sessions on coping that we have proposed could train foster carers to engage with children more effectively, especially at difficult times, and could thus enhance the sustainability of the foster care service.

## Supporting information

S1 Supporting Information English questionnaire. (DOCX)

S2 Supporting Information Romanian questionnaire. (DOCX)
