# Supplementary material for: Reasons to care: Personal motivation as a key factor in the practice of the professional foster carer in Romania
Source: PLoS One. 2019 Sep 18;14(9):e0222716. doi: 10.1371/journal.pone.0222716 (PMC6750651; doi:10.1371/journal.pone.0222716)
Supplement: S1 Supporting Information — (DOCX) [file pone.0222716.s001.docx]

# **Questionnaire**

1. **The reason you chose to become a professional foster carer is: (please circle the answer which is most relevant for you)**

- I did not have a job at the time
- I had lost my job
- I was looking for a change in my profession
- I needed some extra income to help my family
- Our biological children had left home
- Out of concern for the wellbeing of the children in foster care
- I regarded it as a call from God (religious reasons)
- I had a childhood full of suffering and I wanted to give someone in a similar situation a better life
- I had lost a child
- Due to medical reasons, we were not able to have our own children
- We tried to adopt but did not succeed
- Other reasons (please fill in) ___________________________________.

**2. Regarding the care of a child in the foster care system, which of the following options represent an opportunity, from your point of view?**

- The possibility to offer a moral model
- The possibility to offer a spiritual model
- Contributing to the formation of the child’s character
- Developing special relationships
- Assisting a child to develop his/her potential

**3. Please indicate other opportunities which you have enjoyed as a professional foster carer**

__________________________________________________________________________________________________________________________________________________________________________________________________________________________________________

**4. Which of the challenges listed below have you encountered during your experience in the foster care system? Please circle the relevant option(s).**

A. The need to discipline the child(ren)

B. Change of negative behavior

C. Offensive attitudes from the child

D. Attachment disorder

E. Alcohol / Tobacco / Drug use

F. Caring for a child who experiences rejection or bullying in social groups

G. Others___________________________________________________________________

**5. Have you encountered specific dillemmas (not being sure how to act), when caring for the foster child(ren)? If so, please give some examples of such dilemmas** ___________________________________________________________________________________________________________________________________________________________

**6. What kind of dilemmas have you encountered most often?**

A. Ethical dilemmas

B. Dilemmas related to the future separation from the child

C. Religious dilemmas

D. Dilemmas related to the child’s education

E. Dilemmas related to the child’s health

F. Other types of dilemmas:____________________________________________________

**7. Do you believe that sharing moral-Christian values is important as part of the education of the children in your care? Please tick the appropriate box**

| YES |  | NO |  | I DON’T KNOW |  |
| --- | --- | --- | --- | --- | --- |

**8. On a scale of 1 to 10 (where 1 is the smallest and 10 largest) how much do you want your children to have the same moral values as yourself? Please circle.**

1______2______3______4______5______6______7______8______9______10

**9. On a scale of 1 to 5, where 1 is the smallest and 5 largest, how hard are you affected by a child's refusal to follow your indications on moral-Christian values?**

1__________2___________3____________4______________5

**10. What do you do if the child does not listen to your moral-Christian guidance?**

 I do not do anything, I give up trying

 I am often unsure how to act

 I punish him/her

 I continue making an effort to offer a good model

 I continue trying to explain, at another time

 Another option. Please fill in:___________________________________________

**11.** **What do you do if you want to teach the child about prayer, for instance? Please explain briefly** ____________________________________________________________________________________________________________________________________________________

**12. How long have you been a foster carer? Please fill in** __________________.

**13. Your age:**:_______

**14. What is the age of the child(ren) in your care? Please fill in**

______________________________________________________________________________

**15. Do you belong to a religious denomination? Which one**

- Orthodox
- Catholic
- Reformed
- Greek-Catholic
- Pentecostal
- Baptist
- Other:______________________________

**16. What kind of environment do you live in:**

- Urban
- Rural

**Thank you very much for your time!**
