# Supplementary material for: Reasons to care: Personal motivation as a key factor in the practice of the professional foster carer in Romania
Source: PLoS One. 2019 Sep 18;14(9):e0222716. doi: 10.1371/journal.pone.0222716 (PMC6750651; doi:10.1371/journal.pone.0222716)
Supplement: S2 Supporting Information — (DOCX) [file pone.0222716.s002.docx]

# Chestionar

**1. Motivul pentru care ați ales să deveniți asistent maternal profesionist este următorul: (încercuiți cel mai relevant răspuns pentru dumneavoastră)**

- Nu aveam un loc de muncă
- Îmi pierdusem locul de muncă
- Îmi doream o schimbare în domeniul profesional
- Aveam nevoie de un venit în plus pentru a-mi ajuta familia
- Am rămas siguri/singură după ce copiii biologici au plecat de acasă
- Din preocupare pentru binele copiilor luați în asistență maternală
- Am considerat că este o chemare din partea lui Dumnezeu- motive religioase
- Am avut o copilărie plină de suferință și am vrut să ofer cuiva aflat în aceeași situație o viață mai bună
- Am pierdut un copil
- Nu am reușit din motive medicale să avem proprii copii
- Am dorit să adoptăm, dar nu am reușit
- Alte motive (completați) ___________________________________.

**2. Legat de îngrijirea unui copil aflat în sistemul de asistență maternală, care dintre situațiile de mai jos le considerați oportunități?**

- Posibilitatea de a oferi unui model moral
- Posibilitatea de a oferi un model spiritual
- Participarea la formarea caracterului unui copil
- Formarea unor relații speciale
- Îndreptarea potențialului copilului spre performanță

**3. Enumerați, vă rog și alte oportunități de care v-ați bucurat de-a lungul profesiei dumneavoastră.**

__________________________________________________________________________________________________________________________________________________________________________________________________________________________________________

**4. Care dintre provocările enumerate mai jos, le-ați întâlnit de-a lungul experienței dumneavoastră în îngrijirea copiilor aflați în sistemul de asistență maternală? Încercuiți varianta aleasă.**

**A.** Nevoia de disciplinare a copiilor **B.** Schimbarea comportamentului negativ

**C.** Comportament respingător din partea copilului **D.** Atașamentul defectuos

**E.** Consumul de alcool/tutun/droguri

**F.** Îngrijirea unui copil care experimentează respingerea sau bullying în grupurile sociale

**G.** Altele___________________________________________________________________

**5. Ați întâmpinat anumite dileme (nefiind siguri cum ar trebui procedat) în îngrijirea copilului? Dacă da, oferiți-ne câteva exemple de dileme întâmpinate**:_________________________________________________________________________________________________________________________________________________

**6. Din care categorie fac parte dilemele cu care v-ați confruntat cel mai des?**

A. Dileme etice B. Dileme legate de momentul separării de copil

C. Dileme pe plan religios D. Dileme legate de educația copilului

E. Dileme legate de starea de sănătate a copilului

D. Altă categorie:_______________________________________________________________

**7. Considerați că transmiterea valorilor moral-creștine este importantă în procesul de educare al copiilor aflați în grijă. Bifați în căsuță răspunsul ales.**

| DA |  | NU |  | NU ȘTIU |  |
| --- | --- | --- | --- | --- | --- |

**8. Pe o scală de la 1 la 10, (unde 1 este cel mai mic, iar 10 cel mai mare) cât de mult vă doriți ca copiii pe care îi aveți în grijă să aibă aceleași valori morale ca și dumneavoastră? Încercuiți.**

1______2______3______4______5______6______7______8______9______10

**9. Pe o scală de la 1 la 5, unde 1 este cel mai mic, iar 5 cel mai mare, cât de tare sunteți afectat/ă de refuzul unui copil de a urma indicațiile dumneavoastră în ce privește valorile moral-creștine?**

1__________2___________3____________4______________5

**10. Dacă copilul nu ascultă de îndrumările dumneavoastră moral-creștine cum procedați?**

- Nu mai fac nimic, mă dau bătut/ă.
- Modul de acționare constituie o dilemă de multe ori pentru mine
- Îl pedepsesc
- Continui prin propriul meu exemplu să ii ofer în continuare un model bun
- Continui să ii explic într-o altă perioadă
- Altă opțiune, completați._______________________________

**11. Cum procedați în cazul în care doriți să-l învățați pe copilul aflat în îngrijire despre rugăciune, spre exemplu? Completați scurt**. _______________________________________________________________________________________________________________________________________________________

**12. De cât timp profesați**? Completați. __________________.

**13. Vârsta dumneavoastră**:_______ ani.

**14. Ce vârstă are copilul/copiii aflați în grija dumneavoastră? Completați cu cifre.**

______________________________________________________________________________

**15. Aparțin religiei:**

- Ortodoxă
- Catolică
- Reformată
- Greco-catolică
- Penticostală
- Baptistă
- Altă religie:______________________________

**16. Mediul de proveniență:**

- Urban
- Rural

**Mulțumesc foarte mult pentru timpul acordat!**
